# *Leptospira interrogans* Secreted Proteases Degrade Extracellular Matrix and Plasma Proteins From the Host

**DOI:** 10.3389/fcimb.2018.00092

**Published:** 2018-03-27

**Authors:** Ludmila B. da Silva, Milene C. Menezes, Eduardo S. Kitano, Ana K. Oliveira, Afonso G. Abreu, Gisele O. Souza, Marcos B. Heinemann, Lourdes Isaac, Tatiana R. Fraga, Solange M. T. Serrano, Angela S. Barbosa

**Affiliations:** ^1^Laboratory of Bacteriology, Butantan Institute, São Paulo, Brazil; ^2^Special Laboratory of Applied Toxinology, Center of Toxins, Immune-Response and Cell Signaling, Butantan Institute, São Paulo, Brazil; ^3^Brazilian Biosciences National Laboratory, Brazilian Center for Research in Energy and Materials, Campinas, São Paulo, Brazil; ^4^Postgraduation Program in Parasitic Biology, CEUMA University, São Luís, Brazil; ^5^Postgraduation Program in Health Sciences, Federal University of Maranhão, São Luís, Brazil; ^6^Department of Preventive Veterinary Medicine and Animal Health, School of Veterinary Medicine and Animal Science, University of São Paulo, São Paulo, Brazil; ^7^Department of Immunology, Institute of Biomedical Sciences, University of São Paulo, São Paulo, Brazil

**Keywords:** *Leptospira*, secreted proteins, extracellular matrix, plasma proteins, host invasion

## Abstract

Leptospires are highly motile spirochetes equipped with strategies for efficient invasion and dissemination within the host. Our group previously demonstrated that pathogenic leptospires secrete proteases capable of cleaving and inactivating key molecules of the complement system, allowing these bacteria to circumvent host's innate immune defense mechanisms. Given the successful dissemination of leptospires during infection, we wondered if such proteases would target a broader range of host molecules. In the present study, the proteolytic activity of secreted leptospiral proteases against a panel of extracellular matrix (ECM) and plasma proteins was assessed. The culture supernatant of the virulent *L. interrogans* serovar Kennewicki strain Fromm (LPF) degraded human fibrinogen, plasma fibronectin, gelatin, and the proteoglycans decorin, biglycan, and lumican. Interestingly, human plasminogen was not cleaved by proteases present in the supernatants. Proteolytic activity was inhibited by 1,10-phenanthroline, suggesting the participation of metalloproteases. Moreover, production of proteases might be an important virulence determinant since culture-attenuated or saprophytic *Leptospira* did not display proteolytic activity against ECM or plasma components. Exoproteomic analysis allowed the identification of three metalloproteases that could be involved in the degradation of host components. The ability to cleave conjunctive tissue molecules and coagulation cascade proteins may certainly contribute to invasion and tissue destruction observed upon infection with *Leptospira*.

## Introduction

*Leptospira* are long, thin, spiral-shaped, and highly motile Gram-negative bacteria. These spirochetes can be either non-pathogenic free-living organisms or pathogenic, having the potential to cause disease in animals and humans. During infection, leptospires invade multiple organs and tissues, and damage the endothelial linings of the small blood vessels. Severe cases of leptospirosis are characterized by multiple symptoms that may include vascular injury, thrombocytopenia, jaundice, kidney failure, pulmonary hemorrhage, and ocular manifestations such as uveitis and conjunctival congestion (Levett, 2001).

Pathogenic leptospires efficiently spread and propagate in susceptible hosts. They are equipped with strategies to modulate the surrounding microenvironment in the host, including mechanisms to circumvent host's immune responses (Meri et al., 2005; Barbosa et al., 2009). The secretion of proteases that inactivate essential host proteins is an important tool used by diverse microorganisms during the colonization process. *Leptospira* is no exception to this phenomenon, since pathogenic strains have been shown to secrete proteases capable of degrading several proteins of the complement cascade, contributing to serum resistance (Fraga et al., 2014; Amamura et al., 2017). Many bacterial proteolytic enzymes can be considered virulence factors. By hydrolyzing diverse proteinaceous substrates of the host, bacterial proteases play a crucial role in colonization and spreading, allowing evasion of innate immune responses and contributing to disruption of tissue integrity.

The knowledge on the mechanisms underlying tissue damage during leptospirosis is still limited. Like other bacteria, leptospires cross epithelial and endothelial host barriers to get access to target organs. Adhesion to and degradation of the extracellular matrix (ECM), notably of basement membranes, are certainly required for invasion. To date, few studies have been conducted to experimentally identify and characterize leptospiral proteases. It has been shown that a number of *Leptospira* serovars produce multiple gelatinases ranging from 32 to 240 kDa (Madathiparambil et al., 2011). One of them, named ColA, was further characterized and was shown to hydrolyze different types of collagen. A *colA* mutant strain displayed attenuated transcytosis through human embryonic kidney cell lineage HEK293 and human umbilical vein endothelial cell (HUVEC) monolayers, and reduced virulence in the hamster model of infection. A reduced number of bacteria in organs of animals infected with the mutant strain was also observed (Kassegne et al., 2014). Leptallo I, a protease that belongs to the M23 family, displays proteolytic activity against elastin. Leptallo I was shown to be secreted to the culture medium during leptospiral growth, and IgG antibodies recognizing the protein could be detected in the sera of patients with laboratory-confirmed leptospirosis (Hashimoto et al., 2013).

In a previous work, we reported the secretion of proteases by pathogenic *Leptospira* as a novel complement evasion mechanism displayed by these spirochetes (Fraga et al., 2014). Given the rapid and successful dissemination of leptospires during infection, we can assume that such proteases target a broader range of host molecules. As such, the purpose of this work was to evaluate the proteolytic activity of secreted leptospiral proteases against a panel of ECM and plasma proteins.

## Materials and methods

### Proteins, antibodies, and plasma

Fibrinogen, plasminogen, thrombin and fibronectin from human plasma, laminin from basement membrane Engelbreth-Holm-Swarm (EHS) murine sarcoma, and decorin from bovine articular cartilage were purchased from Sigma-Aldrich. Gelatin and Matrigel from EHS murine sarcoma were purchased from Difco and BD Biosciences, respectively, and the recombinant proteoglycans lumican (human), and biglycan (human) were purchased from R&D Systems. Rabbit anti-human fibrinogen was from Sigma-Aldrich. Depletion of albumin from human plasma was performed according to Subramanian (1984).

### *Leptospira* strains

The *Leptospira* strains used were *L. biflexa* serovar Patoc strain Patoc I, *L. biflexa* serovar Andamana strain CH11, *L. interrogans* serovar Kennewicki strain Fromm (LPF), *L. interrogans* serovar Copenhageni strain 10A, *L. interrogans* serovar Pomona strain Pomona, *L. kirshneri* serovar Cynopteri strain 3522 C, and *L. noguchi* serovar Panama CZ 214. Virulence of LPF was maintained by iterative passages in hamsters. Infected animals become acutely ill and present symptoms such as jaundice, uveitis, light sensitivity, prostration, loss of appetite. They usually die on day 5 post-infection. Bacteria were cultured for 7 days in modified Ellinghausen-McCullough-Johnson-Harris (EMJH) at 29°C under aerobic conditions as previously described (Barbosa et al., 2010). The leptospires were then washed, counted by dark-field microscopy, and 1 × 10^9^ bacteria resuspended in PBS (pH 7.4) were allowed to secrete proteins for 4 h at 37°C, thus simulating responses to temperature upshift observed during infection of mammalian hosts (Fraga et al., 2014). Protein concentration was determined using the BCA Kit (Pierce).

### Degradation of plasma and ECM molecules

Fibrinogen (15 μg), plasminogen (5 μg), fibronectin (5 μg), thrombin (1 μg), laminin (5 μg), matrigel (5 μg), decorin (2 μg), biglycan (2 μg), and lumican (2 μg) were incubated with culture supernatants from different *Leptospira* strains (0.5 μg) at 37°C for the indicated time points. A sample of each substrate was also incubated without culture supernatants for 18 h under identical conditions. Reactions were stopped by adding Laemmli sample buffer (60 mM Tris-Cl pH 6.8, 2% SDS, 10% glycerol, 5% β-mercaptoethanol, 0.01% bromophenol blue) and subjected to SDS-PAGE. Polyacrylamide gels were subsequently stained. Degradation of fibrinogen in plasma was assessed as follows. Albumin-depleted human plasma (15 μg) was incubated with LPF supernatant (0.01–0.5 μg) for 2 h at 37°C and then submitted to SDS-PAGE followed by electroblotting onto nitrocellulose membrane. Cleavage products were detected using rabbit anti-human fibrinogen antibodies, followed by peroxidase-conjugated anti-rabbit antibodies. Positive signals were detected by enhanced chemiluminescence (West Pico, Pierce). To assess the class of proteases involved in the degradation of ECM and plasma proteins, leptospiral supernatants were preincubated with inhibitors of serine (5 mmol/L phenylmethylsulfonyl fluoride), metallo- (5 mmol/L 1,10-phenanthroline), cysteine (28 μmol/L E-64) or aspartyl (5 μmol/L pepstatin) proteases for 30 min before the addition of the substrate.

### Gelatin zymography

To assess gelatinolytic enzymatic activity in *Leptospira* culture supernatants zymography using gelatin was performed. Gelatin (1 mg/mL) was copolymerized with 12% w/v acrylamide, 0.3% bisacrylamide and 0.375 M Tris-HCl (pH 8.8). Samples (0.5 μg of proteins from culture supernatants) were mixed with non-reducing SDS-PAGE sample buffer (40 mM Tris HCl pH 6.8, 1% SDS, 2% glycerol and 0.01% bromophenol blue) and applied to gels. After electrophoresis, the gels were incubated for 30 min at room temperature on a rotary shaker in 50 mM Tris-HCl, pH 7.4, containing 2.5% Triton X-100. Excess Triton X-100 was removed upon washes with deionized water and then the gels were incubated in a buffer containing 0.05 M Tris–HCl, pH 8.0, 0.15 M NaCl, 0.01 M CaCl_2_, 0.02% CHAPS at 37°C for 12 h. The gels were stained with Coomassie blue and destained. Gelatin digestion was identified as clear zones of lysis against a blue background.

### Mass spectrometry and protein identification

*L. interrogans* (LPF) and *L. biflexa* (Patoc I) (10^9^ bacteria) cultured in EMJH medium at 29°C were pelleted by centrifugation at 3,200 × g for 15 min, and washed twice with PBS, pH 7.4. The bacteria were resuspended in PBS and incubated at 37°C for 4 h to allow secretion of proteins, and centrifuged at 3,200 × g for 10 min. Supernatants were collected, passed through a 0.22 μm filter (Merck Millipore), and concentrated using Amicon Ultra centrifugal filters (3,000 MWCO; Merck Millipore).

For the identification of *Leptospira* secreted proteins, 30 μg of proteins derived from LPF and Patoc I culture supernatants (three biological replicates) were processed by the Filter Aided Sample Preparation (FASP) procedure (Wiśniewski et al., 2009), using 10 kDa Microcon filtration devices (Millipore). The resulting protein mixture was diluted to 0.06 mL of 0.05 M NH_4_HCO_3_ and trypsin (Sigma) was added at a 1:50 enzyme-to-substrate ratio, and submitted to incubation at 37°C for 18 h. Peptide samples were desalted using StageTips C18 according to Rappsilber et al. (2007).

Each peptide mixture (5 μL) was injected into a 5 cm Jupiter® C-18 trap column packed in-house (Phenomenex; 100 μm I.D. × 360 μm O.D.) using a EASY nanoLCII system (Thermo Fisher Scientific) coupled to an LTQ-Orbitrap Velos mass spectrometer (Thermo Fisher Scientific). Chromatographic separation of tryptic peptides was performed on a 10-cm long column (75 μm I.D. × 360 μm O.D.) packed in-house with 5 μm Aqua® C-18 beads (Phenomenex). Peptides eluted with a linear gradient of 5–35% acetonitrile in 0.1% formic acid (solution B) at 200 nL/min in 75 min, 35–80% B in 15 min, 80% B for 7 min, back to 5% in 1 min and 5% B for 22 min. Spray voltage was set to 2.4 kV and the mass spectrometer was operated in data dependent mode, in which one full MS scan was acquired in the *m*/*z* range of 300–1,700 followed by MS/MS acquisition using collision induced dissociation of the twelve most intense ions from the MS scan. MS spectra were acquired in the Orbitrap analyzer at 60,000 resolutions (at 400 m/z) whereas the MS/MS scans were acquired in the linear ion trap. Isolation window, activation time and normalized collision energy were set to, respectively, 3 m/z, 10 ms and 35%. A dynamic peak exclusion was applied to avoid the same *m/z* of being selected for the next 60 s. Three technical replicates were performed.

LTQ-Orbitrap Velos raw data were searched using the Andromeda algorithm (Cox et al., 2011) at the MaxQuant environment (Cox and Mann, 2008; version 1.5.0.0), using a target database restricted to the genus *Leptospira* (UniProt database containing 366,195 protein sequences, downloaded on Nov 30, 2015) to which a set of reverse sequences were added (decoy dataset), with a parent and fragment mass tolerance of 10 ppm and 0.5 Da, respectively. Carbamidomethylation was set as fixed modification, and oxidation of methionine, protein N-acetylation and deamidation of asparagine or glutamine were specified as variable modifications. Two missed cleavages were allowed and the minimal length required for a peptide was seven amino acids. One unique peptide was required for protein identifications. The false discovery rate (FDR) at peptide and protein level was adjusted to 1%. The output of the search was processed using Perseus (Tyanova et al., 2016; version 1.5.0.15). Contaminants, reverse decoy proteins, and proteins identified only by a modification site were removed from search. Only proteins identified in two of three biological replicate data sets were accepted.

## Results

### Degradation of ECM and plasma proteins by *L. interrogans*

Degradation of structural and soluble host molecules contributes to bacterial dissemination facilitating invasion and colonization of target organs. In this work, we evaluated the proteolytic activity of leptospiral secreted proteases against ECM and plasma proteins. Secreted proteins were obtained upon incubation of leptospires at 37°C in PBS because it has been previously shown that proteolytic activity against host's complement molecules is clearly observed under these conditions (Fraga et al., 2014).

**Figure 1:**
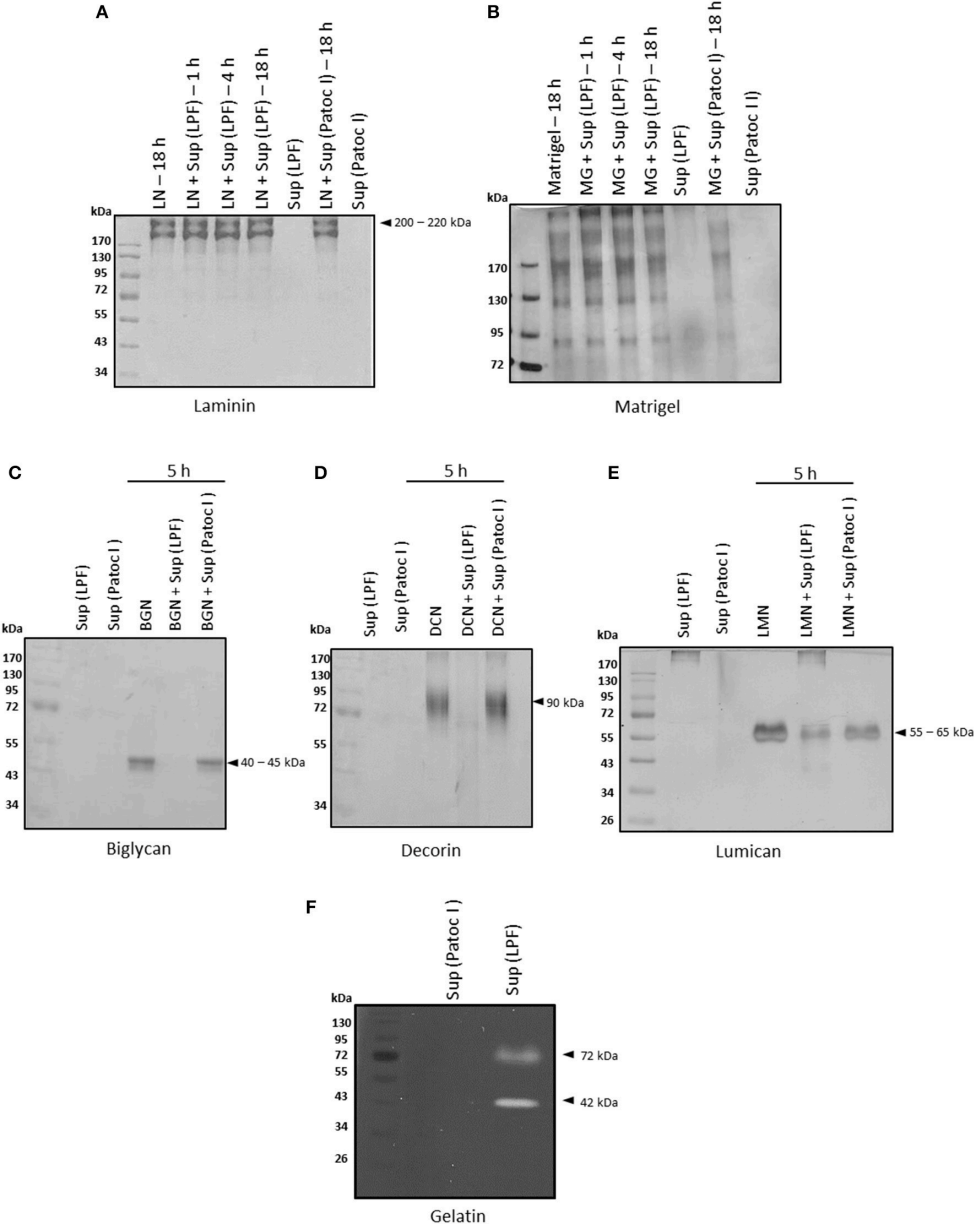
Degradation of ECM proteins by *Leptospira interrogans* secreted proteins. **(A)** Laminin (5 μg), **(B)** matrigel (5 μg), **(C)** biglycan (2 μg), **(D)** decorin (2 μg), and **(E)** lumican (2 μg) were incubated with supernatants of pathogenic *Leptospira interrogans* serovar Kennewicki strain Fromm (LPF) or saprophytic *Leptospira biflexa* serovar Patoc strain Patoc I (0.5 μg of total secreted proteins) at 37°C for the indicated time points. Cleavage products were evaluated by SDS-PAGE under reducing conditions. Gels were silver stained. **(F)** Proteins present in both supernatants (0.5 μg of total secreted proteins) were submitted to zymography on a 12% SDS-polyacrylamide gel copolymerized with gelatin and Coomassie Brilliant Blue stained. LN, laminin; MG, matrigel; BGN, biglycan; DCN, decorin; LMN, lumican; Sup (LPF), supernatant of *Leptospira interrogans* serovar Kennewicki strain Fromm; Sup (Patoc), supernatant of *Leptospira biflexa* serovar Patoc strain Patoc I.

Basement membranes, predominantly composed of laminin and collagen IV, are specialized extracellular matrices that separate the epithelium and endothelium from underlying connective tissue. Since degradation of basement membrane components is crucial for invasion, we first assessed proteolytic activity of *Leptospira* culture supernatants against laminin and matrigel. Under our experimental conditions, laminin and matrigel were not degraded by proteins from leptospiral supernatants (Figures 1A,B).

Proteoglycans, heavily glycosylated proteins, are also major components of extracellular matrices. They fill spaces in the ECM by bridging different macromolecules and provide a highly hydrated gel-like microenvironment (revised in Chagnot et al., 2012). Degradation of small leucine-rich proteoglycans (SLRP) by proteases present in leptospiral culture supernatants was also assessed. At an enzyme/substrate ratio of 1: 4, i.e., 0.5 μg of supernatant: 2 μg of proteoglycan, decorin and biglycan were fully degraded after incubation with the virulent strain LPF supernatant whereas lumican was partially degraded (Figures 1C–E). At an enzyme/substrate ratio of 1: 10, proteoglycan hydrolysis was much less pronounced (data not shown).

In addition, proteases of approximately 72 and 42 kDa with gelatinolytic activity were detected in the presence of the LPF supernatant, as revealed by gel-zymography (Figure 1F). Regardless of the substrate used, no proteolytic activity was observed upon incubation with the saprophytic strain Patoc I supernatant (Figure 1).

**Figure 2:**
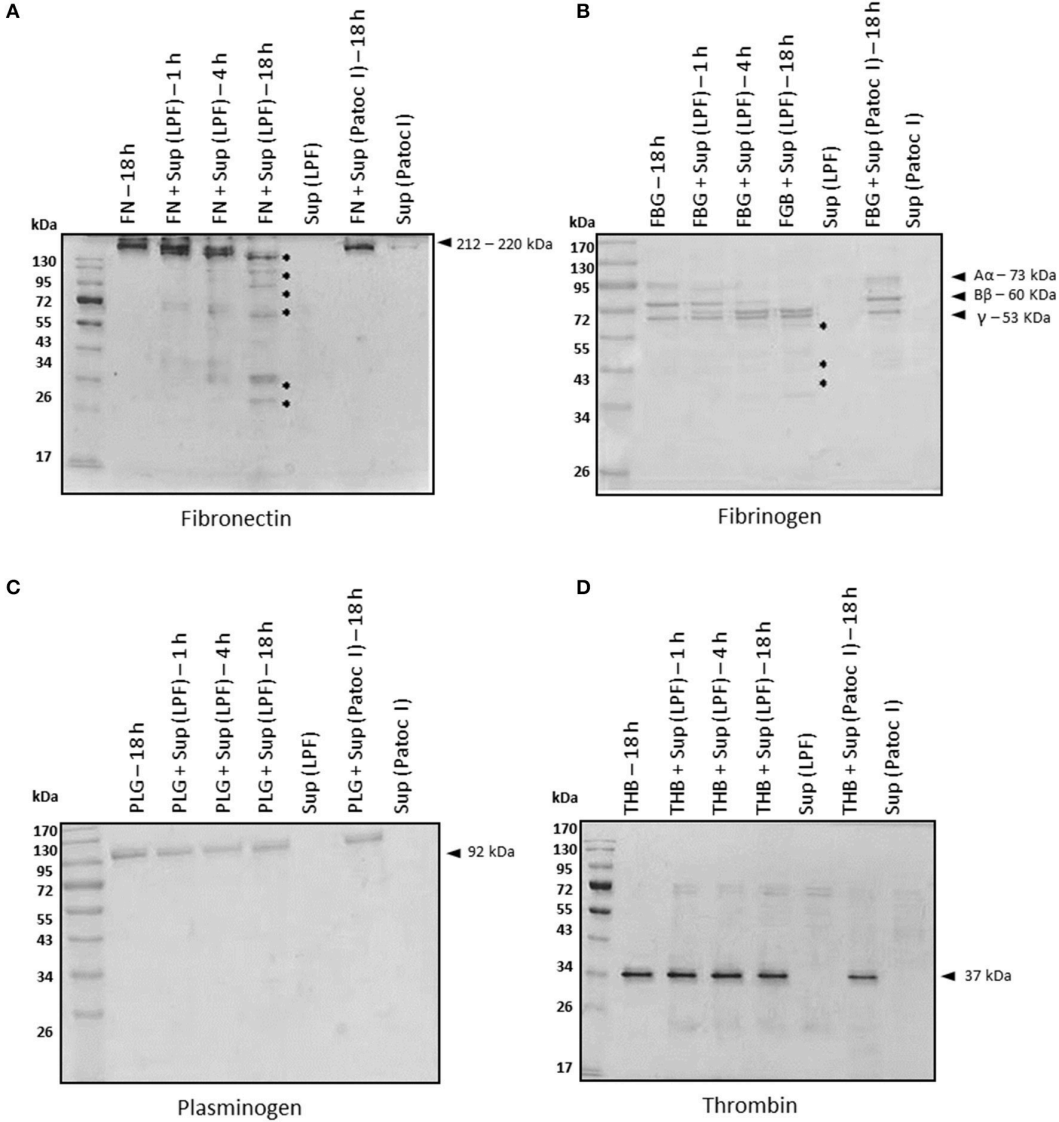
Degradation of plasma proteins by *Leptospira interrogans* secreted proteases**. (A)** Plasma fibronectin (5 μg), **(B)** fibrinogen (15 μg), **(C)** plasminogen (5 μg), and **(D)** thrombin (1 μg) were incubated with supernatants of pathogenic *Leptospira interrogans* serovar Kennewicki strain Fromm or saprophytic *Leptospira biflexa* serovar Patoc strain Patoc I (0.5 μg of total secreted proteins) at 37°C for the indicated time points. Cleavage products were evaluated by SDS- polyacrylamide gel under reducing conditions. Gels were silver stained. FN, plasma fibronectin; FBG, fibrinogen; PLG, plasminogen; THB, thrombin; Sup (LPF), supernatant of *Leptospira interrogans* serovar Kennewicki strain Fromm; Sup (Patoc), supernatant of *Leptospira biflexa* serovar Patoc strain Patoc I.

Blood coagulation proteins such as fibrinogen, plasminogen, and thrombin, as well as plasma fibronectin, were also tested as substrates for leptospiral proteases. Plasma fibronectin and fibrinogen were time-dependently degraded by LPF proteases whereas no degradation occurred in the presence of Patoc I supernatant, even after 18 h of incubation (Figures 2A,B). Fibronectin fragments ranging from 25 to 170 kDa were generated upon a prolonged incubation period (Figure 2A). Smaller fibrinogen fragments of 25–45 kDa were also produced as a consequence of both α- and β-chain degradation (Figure 2B). An enzyme/substrate ratio of 1: 30, i.e., 0.5 μg of supernatant: 15 μg of purified fibrinogen, seems to be the minimum required ratio for complete degradation of both α and β chains (Supplementary Figure 1), and this condition was then chosen for subsequent assays. Proteolytic activity using albumin-depleted human plasma further confirmed fibrinogen degradation by proteinases present in the LPF supernatant (Supplementary Figure 1). Reduced amounts of leptospiral proteases can degrade fibrinogen. By contrast, plasminogen and thrombin were not susceptible to leptospiral proteases, as depicted in Figures 2C,D.

### Metalloproteases secreted by *L. interrogans* degrade proteoglycans, plasma fibronectin and fibrinogen

**Figure 3:**
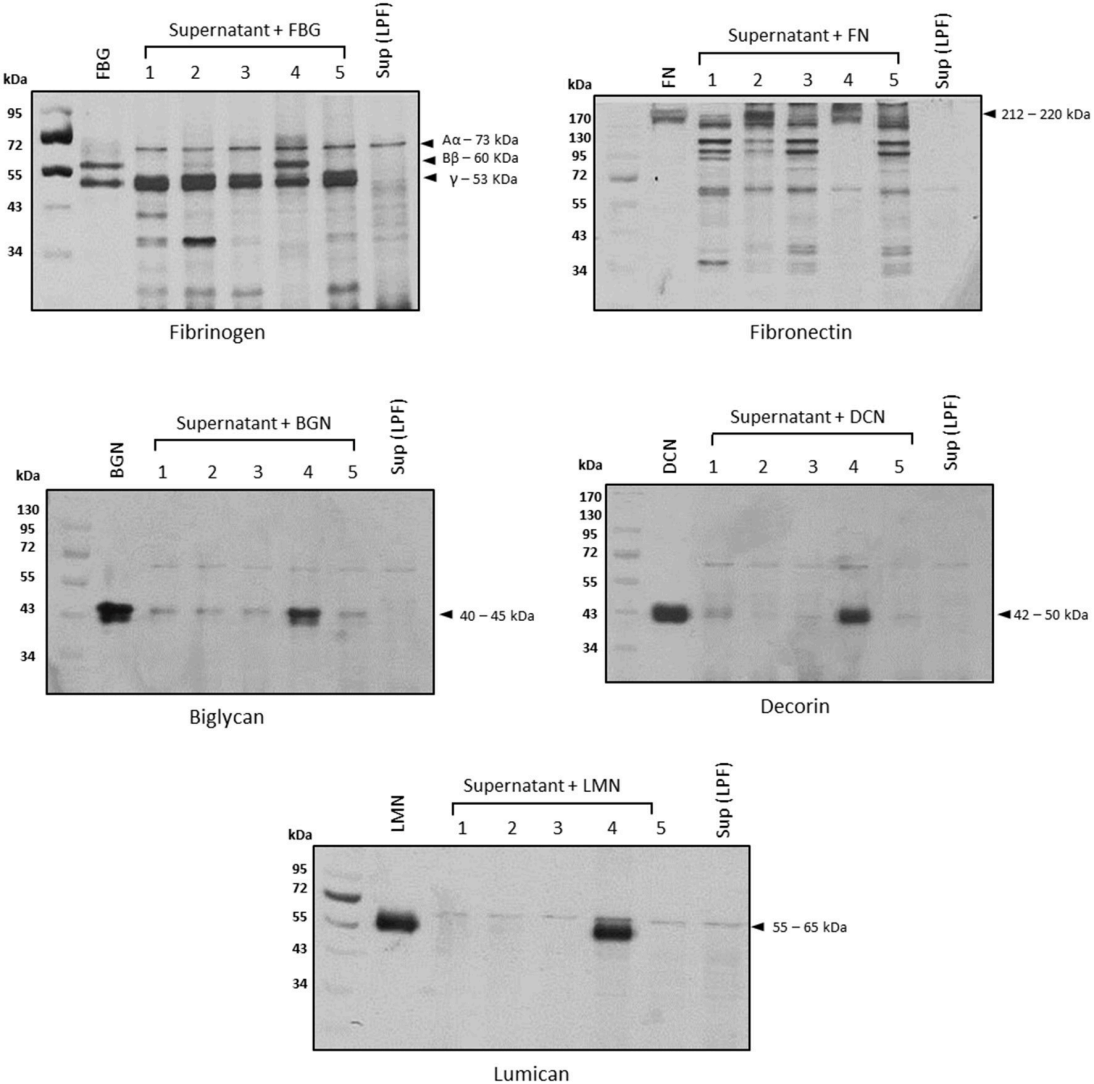
Proteolytic activities are inhibited by 1,10-phenanthroline. Before the addition of each substrate, the supernatant of pathogenic *Leptospira interrogans* serovar Kennewicki strain Fromm (0.5 μg of total secreted proteins) was incubated with inhibitors of serine proteases (5 mmol/L PMSF; lane 2), cysteine proteases (28 μmol/L E-64; lane 3), metalloproteases (5 mmol/L 1,10-phenanthroline; lane 4), or aspartyl proteases (5 μmol/L pepstatin; lane 5) for 30 min at room temperature. Substrates were added and incubations proceeded for 18 h. Cleavage products were analyzed by SDS- polyacrylamide gel under reducing conditions. Gels were silver stained. FBG, fibrinogen; FN, plasma fibronectin; BGN, biglycan; DCN, decorin; LMN, lumican; Sup (LPF), supernatant of *Leptospira interrogans* serovar Kennewicki strain Fromm.

To assess the class(es) of proteases involved in the degradation of host molecules, LPF supernatant was pretreated with inhibitors of serine, metallo-, cysteine, or aspartyl proteases before the addition of each substrate. In all cases, only 1,10-phenanthroline could fully inhibit the proteolytic activities, strongly suggesting the involvement of metalloproteases in this process (Figure 3, lane 4).

### Proteolytic activity against host molecules correlates with *L. interrogans* virulence

**Figure 4:**
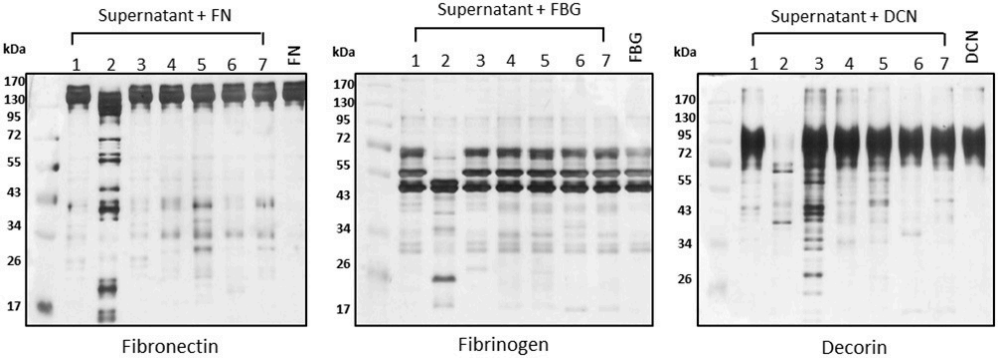
Proteolytic activity correlates with virulence. Supernatants of (1) *L. biflexa* serovar Patoc strain Patoc I, (2) *L. interrogans* serovar Kennewicki strain Fromm (LPF), (3) *L. biflexa* serovar Andamana strain CH11, (4) *L. interrogans* serovar Copenhageni strain 10A, (5) *L. interrogans* serovar Pomona strain Pomona, (6) *L. kirshneri* serovar Cynopteri strain 3522 C, (7) *L. noguchi* serovar Panama CZ 214 (0.5 μg of total secreted proteins) were incubated with plasma fibronectin (5 μg), fibrinogen (15 μg), and decorin (2 μg) for 18 h h at 37°C. Cleavage products were analyzed by SDS- polyacrylamide gel under reducing conditions. Gels were silver stained. FN, plasma fibronectin; FBG, fibrinogen; DCN, decorin. Lane 8: controls (purified substrates).

To assess the proteolytic potential of additional *Leptospira* strains, we used a panel of seven *Leptospira* strains including one virulent (*L. interrogans* serovar Kennewicki strain Fromm, also called “LPF”), two saprophytes (*L. biflexa* serovar Patoc strain Patoc I and *L. biflexa* serovar Andamana strain CH11), and four culture-attenuated (*L. interrogans* serovar Copenhageni strain 10A, *L. interrogans* serovar Pomona strain Pomona, *L. kirshneri* serovar Cynopteri strain 3522 C and *L. noguchi* serovar Panama CZ 214). Strains LPF and Patoc I were used in the previous assays presented in Figures 1–3. Interestingly, proteolytic activity was only observed in the presence of the virulent strain LPF supernatant, thus indicating that production of proteases might be an important virulence determinant (Figure 4). Culture-attenuated or saprophytic *Leptospira* strains did not display a significant proteolytic activity against fibronectin, decorin or fibrinogen (Figure 4). Culture supernatants from the seven *Leptospira* strains used in this study were the same as those used in Fraga et al. (2014).

### Identification of secreted proteins from LPF and patoc I strains

All secreted proteins identified in the LPF and Patoc I strains are shown in Supplementary Table 1. A total of 236 and 161 unique proteins were identified, respectively, in the LPF and in the Patoc I exoproteomes (Supplementary Tables 2, 3). Fifty proteins were detected in both strains (Supplementary Table 4). A search aiming to find proteases unique to the LPF exoproteome allowed identification of six peptidases, out of which three are metalloproteases (Table 1). One of them, a 52 kDa protein belonging to the M43 family, harbors a pappalysin-1 domain and contains a HEXXHXXGXXH zinc-dependent active site. Human pappalysin-1 is described as a secreted metalloprotease which cleaves insulin-like growth factor binding proteins (Oxvig, 2015). This protein also plays a role in bone formation, inflammation, wound healing and female fertility. A role for proteins containing pappalysin-1 domains remains to be exploited in bacteria. Another metalloprotease identified was TldD, a 49.9 kDa protease containing the conserved HEXXH zinc-binding motif. TldD is quite conserved among prokaryotes, and plays a role in degrading unfolded proteins, as well as in the activation and degradation of natural products such as the peptide antibiotic microcin B17, peptide-derived cofactors or toxin-antitoxin modules (Ghilarov et al., 2017). The third metalloproatese identified was the 26.6 kDa methionine aminopeptidase (MAP) known to catalyze the hydrolytic cleavage of the N-terminal methionine from newly synthesized polypeptides. Apparently, neither of the three aforementioned proteases seems to be involved in gelatin hydrolysis since according to our zymography data gelatinolytic activity was associated with proteases of 72 and 42 kDa (Figure 1F). Not infrequently, proteases are synthesized in very small quantities despite being highly efficient in hydrolyzing substrates, what may explain the non-detection of proteases presenting molecular masses of 72 and 42 kDa in our proteomic analysis.

**Table 1:** Proteases identified in the *L. interrogans* serovar Kennewicki strain Fromm (LPF) exoproteome.

**EMBL GenBank**	**Protein**	**Gene**	**Class**
EKO25673.1	Pappalysin-1 domain protein	LEP1GSC104_4629	Metallopeptidase
AAN49383.1	Predicted Zn-dependent protease	*tldD*	Metallopeptidase
AAN49656.2	Methionine aminopeptidase	*map*	Metalloaminopeptidase
EMP06453.1	Papain family cysteine protease	LEP1GSC124_0278	Cysteine peptidase
M3CS93	ATP-dependent protease ATPase subunit HslU	*hslU*	Peptidase
AAN49152.2	ATP-dependent Clp protease proteolytic	*clpP*	Serine endopeptidase

**Figure 5:**
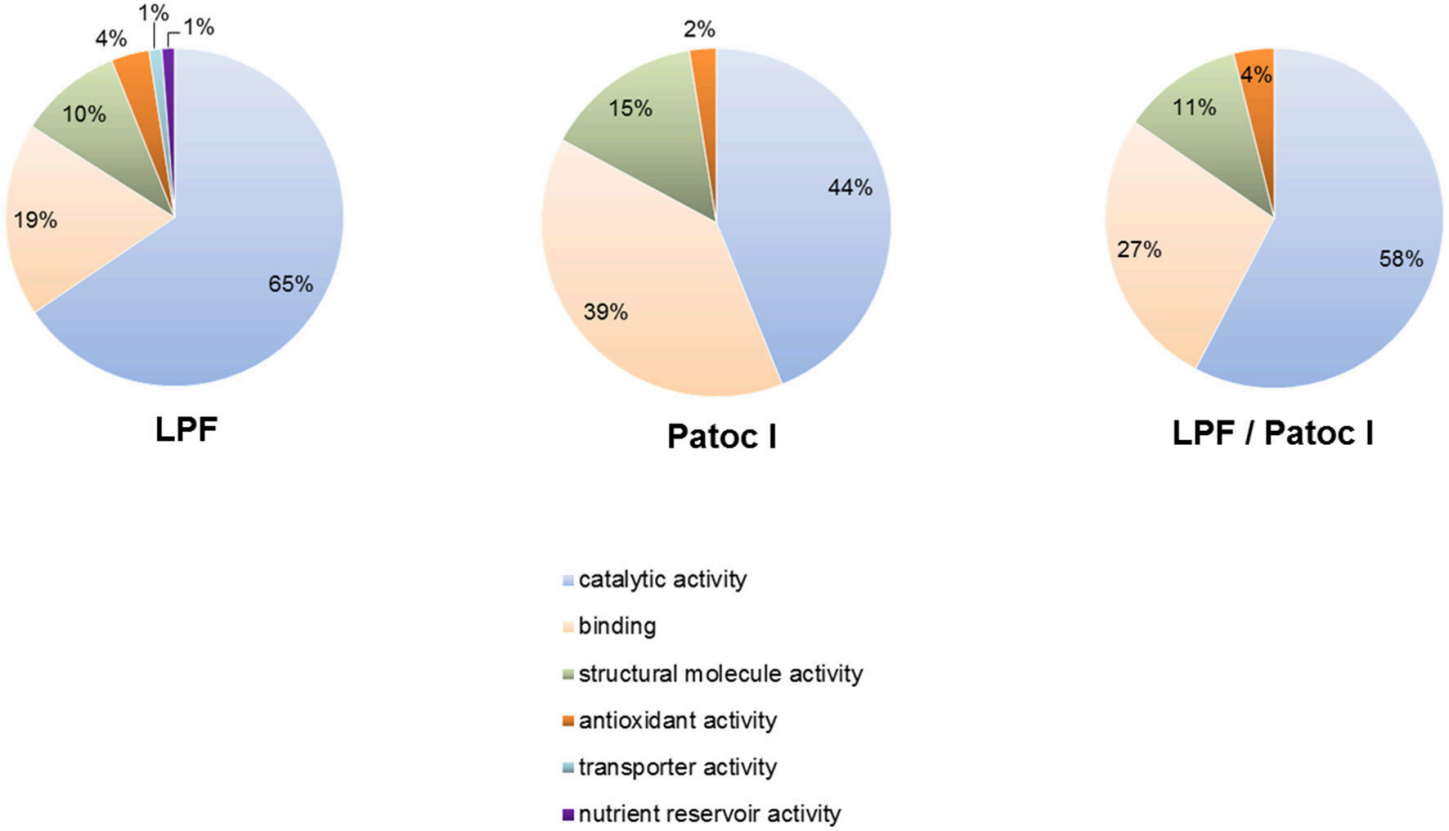
Assignment of secreted proteins to Clusters of Orthologous Groups of proteins (COGs). The proteins identified in *L. interrogans* and *L. biflexa* exoproteomes were classified within the molecular function category. The predicted percentage of proteins in each subcategory is indicated. Pie charts of LPF unique proteins, Patoc I unique proteins and common proteins (LPF/Patoc I) are presented.

LPF and Patoc I secreted proteins were assigned to clusters of orthologous groups (COGs). Under molecular function the proteins were classified into the following categories: catalytic activity, binding, structural molecule activity, antioxidant activity, transporter activity, and nutrient reservoir activity. Proteins involved in catalytic activity and binding were the most represented in both LPF and Patoc I strains (Figure 5).

Using the MoonProt database (Mani et al., 2015), 25 proteins presumed to display moonlighting functions were detected (Supplementary Table 5). These multifunctional proteins play crucial roles in physiological processes, but have been shown to contribute to infection by many pathogens (Henderson, 2017). Housekeeping enzymes, including proteins from the glycolytic pathway, and chaperones are among the moonlighting proteins found in the exoproteomes.

## Discussion

Proteolytic activity targeting host molecules is a key strategy to facilitate the infection process of pathogens. Successful infection is not only achieved by the ability to circumvent host's innate immune defenses, but also by additional mechanisms that may aid bacteria to reach target organs. In this context, secreted proteases are of major importance for the infectious process. They are produced by pathogens including both Gram-positive and Gram-negative bacteria, viruses, and fungi. A number of bacterial proteases contributing to invasion have been described to date. The alkaline protease (AprA), the proteases LasA, LasB, and protease IV from *P. aeruginosa* cause tissue damage during infections by inactivating components of the connective tissue (Schmidtchen et al., 2001). The same applies to Staphopain A (ScpA), aureolysin from *Staphylococcus aureus* and gelatinase (GelE) from *Enterococcus faecalis* which have been shown to degrade collagen, elastin and laminin, and also contribute to immune evasion (Park et al., 2008; Koziel and Potempa, 2013; Senyürek et al., 2014).

Pathogenic *Leptospira* are known to cross tissue barriers and rapidly reach the bloodstream. Target tissues are colonized even before bacterial multiplication (Wunder et al., 2016). Rapid dissemination within the host stems from leptospiral spiral shape associated with a translational motility, allowing them to efficiently swim in viscous gel-like environments (revised in Picardeau, 2017). In addition, proteases released during the initial stages of infection may also contribute to leptospiral invasion and immune evasion (Fraga et al., 2014). In this work, proteolytic activity of secreted leptospiral proteinases against some ECM components and human plasma proteins was evaluated. Proteases present in the culture supernatant of the virulent LPF strain degraded the small leucine-rich proteoglycans decorin, biglycan and lumican.

Ubiquitously distributed in the connective tissue, decorin interacts with multiple ECM compounds, being essential for matrix integrity. It is also involved in collagen fibrillogenesis, wound repair, angiostasis, tumor growth, and autophagy (Gubbiotti et al., 2016). Biglycan, another SLRP, is present in large quantities in skeletal and hard tissues (Wadhwa et al., 2004), but is also found in the kidneys (Schaefer et al., 2000), liver (Högemann et al., 1997), and skin (Fleischmajer et al., 1991). Like decorin and biglycan, lumican plays important roles in ECM organization and modulates biological processes, including regulation of collagen assembly into fibrils (McEwan et al., 2006; Schaefer and Iozzo, 2008; Stamov et al., 2013), and regulation of apoptosis, cell growth, invasion and angiogenesis (revised in Nikitovic et al., 2014). Interestingly, it has been shown that *L. interrogans* binds to proteoglycans expressed by mammalian cells (Breiner et al., 2009). Glycosaminoglycans (GAGs), covalently linked to proteoglycan core proteins, are the primary targets for leptospiral adhesion, and chondroitin sulfate B is the preferred GAG used for binding (Breiner et al., 2009). As decorin and biglycan are chondroitin sulfate/dermatan sulfate proteoglycans, we can speculate that these GAGs may serve as a platform for *Leptospira* anchoring, and subsequent local degradation of proteoglycan core proteins by bacterial secreted proteases.

On the assumption that basement membranes are also targeted by bacterial proteases, the effect of leptospiral secreted proteases on purified laminin and matrigel was also evaluated. Under our experimental conditions, these two substrates were not degraded. Gelatinolytic activity of *Leptospira* supernatants was evaluated by zymography. Proleotytic activity related to protein bands of approximately 72 kDa and 42 kDa was observed in gels co-polymerized with gelatin. A previous work by Madathiparambil et al. (2011) reported the presence of multiple gelatinases extracted from whole *Leptospira* ranging from 32 to 240 kDa. Some of them, including the ones detected in the present work, may be secreted during infection thus contributing to pathogenesis.

Proteolytic cleavage of plasma fibronectin and fibrinogen was also observed upon incubation with the culture supernatant of *L. interrogans* strain LPF. Purified fibrinogen and fibrinogen in human plasma were dose-dependently degraded by LPF proteases. Fibrinogen, a key clotting protein, plays a crucial role in coagulation and homeostasis. As a consequence of vascular damage, thrombin cleaves fibrinogen into insoluble fibrin, triggering the fibrin network formation, essential for homeostasis. During infection several pathogens produce proteinases that degrade fibrinogen and, as a consequence, repair of injured sites is retarded (revised in Sun, 2006). One example is the treponemal metalloproteinase pallilysin (Tp0751), which in concert with the serine protease Tp0750, contributes to *Treponema pallidum* dissemination by inhibiting coagulation, promoting fibrinolysis, and degrading ECM components (Houston et al., 2011, 2012, 2014). Inflammation, vascular damage and lung hemorrhage are typical clinical manifestations of patients with severe leptospirosis (revised in Murray, 2015). Studies aiming to investigate whether these pathologies may be caused by fibrinogen-degrading proteinases of *Leptospira* are currently underway.

Thrombin and plasminogen, two other coagulation cascade molecules, were also incubated with *Leptospira* supernatants, and were not susceptible to degradation. It has been previously shown that *Leptospira* has multiple receptors for human plasminogen, and that, once bound to the bacterial surface, this host protease zymogen is converted to its active form, plasmin (Verma et al., 2010; Vieira et al., 2010; Nogueira et al., 2013; Wolff et al., 2013; Castiblanco-Valencia et al., 2016; Salazar et al., 2017). Since bound-plasmin(ogen) degrades ECM components and complement molecules *in vitro* (revised in Fraga et al., 2016), binding intact plasminogen may be more beneficial to the bacterium than degrading this zymogen.

By analyzing the proteolytic capacity of a larger number of strains, we found that culture-attenuated *Leptospira* strains failed to cleave ECM components, leading us to conclude that the proteases involved in this process are produced only under virulence conditions. Since 1,10-phenanthroline was an effective degradation inhibitor, it is presumed that metalloproteases were responsible for the majority of the observed proteolytic activity. Following these observations, proteomic analysis was performed in order to identify secreted proteases of virulent *Leptospira* that may be responsible for the degradation of host components. Among the proteases identified, pappalysin-1 domain protein deserves special attention. This metalloproteinase is well conserved among pathogenic *Leptospira* species according to Analysis Basic Local Alignment Search Tool (BLAST) analysis. A human ortholog, pregnancy-associated plasma protein-A (PAPP-A), was initially described as an abundant placental protein detected in pregnant women. Further studies have shown that PAPP-A is expressed in a variety of tissues and is found tightly bound to GAGs on cell surfaces. This secreted metalloproteinase targets mainly insulin-like growth factor binding proteins (Oxvig, 2015). Functional characterization of *Leptospira* pappalysin-1 domain protein will be the subject of future studies of our group.

Furthermore, several moonlighting proteins were identified (Supplementary Table 5), thus endorsing previous exoproteomic data obtained from *Leptospira* culture supernatants (Eshghi et al., 2015). It is worth mentioning that the majority of moonlighting proteins reported by Eshghi and colleagues were also detected in our preparations, and among them are elongation Factor Tu (EF-Tu), enolase, and catalase. EF-Tu and enolase were described as plasminogen binding proteins (Nogueira et al., 2013; Wolff et al., 2013). They also interact with the complement regulators Factor H (EF-Tu) and C4b binding protein (C4BP) (Wolff et al., 2013; Salazar et al., 2017). Catalase is involved in oxidative stress resistance (Eshghi et al., 2012). By displaying additional functions related to interactions with host cells, some moonlighting proteins including glyceraldehyde 3-phosphate dehydrogenase (GAPDH) and fructose-1,6-biphosphate aldolase (FBA) are presumed to contribute to bacterial virulence (Henderson, 2017). However, as their canonical functions are generally associated with essential roles within the cell, and knockouts are generally non-viable, the assumption that they may contribute to bacterial virulence sometimes stems purely from *in vitro* experimentation. In addition, 15 out of 25 moonlighting proteins identified in our study were present in the saprophytic strain Patoc I (Supplementary Table 5), raising concerns about their role in virulence. For the moment, what can be assumed is that moonlighting proteins contribute to expand the functional proteome of a particular bacterium (Henderson, 2017). An obvious question that arises from our observations is how these and other proteins devoid of N-terminal signal sequence or predicted to be exported through a non-classical secretory pathway are directed to the extracellular space. In line with the findings reported by Eshghi et al. (2015), around 60% of the proteins identified in the present study fall into this category. Whether *Leptospira* possesses particular export mechanisms remains an open question.

In conclusion, leptospiral extracellular proteases display proteolytic activity against proteoglycans and plasma proteins. The capacity to degrade host molecules correlates with *Leptospira* virulence, and may contribute to its dissemination potential.

## Author contributions

LS performed most of the experiments and prepared all figures. MM contributed to sample preparation, proteomic data analysis, and aided in interpreting the results. EK contributed to proteomic execution. AO contributed to proteomic data analysis and provided a critical feedback. AA performed some of the experiments. GS provided all *Leptospira* strains used in the study. MH contributed to the analysis and interpretation of the data. LI provided critical feedback and helped shape the research. TF contributed to the design and implementation of the research. SS contributed with reagents, and performed the final proteomic analyses; contributed to the writing of the manuscript. AB conceived the study and was in charge of overall direction and planning, and wrote the manuscript.

### Conflict of interest statement

The authors declare that the research was conducted in the absence of any commercial or financial relationships that could be construed as a potential conflict of interest.

## Supplementary material

Supplementary Figure 1 Dose-dependent cleavage of purified and plasma fibrinogen. *Leptospira interrogans* serovar Kennewicki strain Fromm (LPF) supernatant (0.01–0.5 μg total secreted proteins) was incubated with 15 μg of purified fibrinogen **(A)** or with human plasma containing an equivalent amount of fibrinogen **(B)** for 2 h at 37°C. Cleavage products were analyzed by SDS- polyacrylamide gel under reducing conditions and gels were silver stained **(A)** or submitted to Western blot and detected with anti-human fibrinogen **(B)**. ^*^cleavage products. FBG, fibrinogen.

Supplementary Table 1 Identification of proteins in LPF and Patoc I strains by in solution tripsin digestion and LC-MS-MS analysis.

Supplementary Table 2 Proteins identified only in the LPF strain.

Supplementary Table 3 Proteins identified only in the Patoc I strain.

Supplementary Table 4 Proteins identified in the supernatants of both LPF and Patoc I strains.

Supplementary Table 5 Putative moonlighting proteins identified in LPF and Patoc I culture supernatants.
