# Supplementary figures and images for: Leptospira interrogans Secreted Proteases Degrade Extracellular Matrix and Plasma Proteins From the Host
Source: Front Cell Infect Microbiol. 2018 Mar 27;8:92. doi: 10.3389/fcimb.2018.00092 (PMC5881292; doi:10.3389/fcimb.2018.00092)

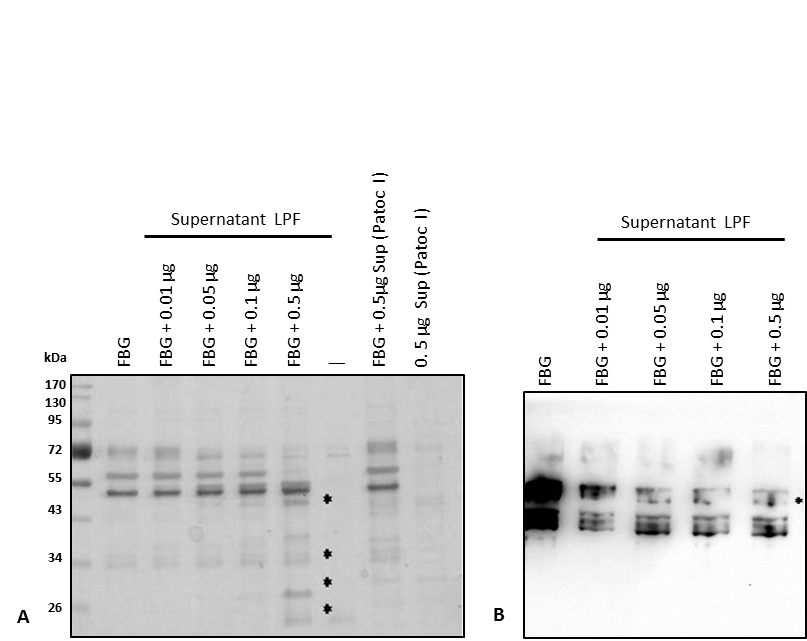

Supplement: Supplementary Figure 1 — Dose-dependent cleavage of purified and plasma fibrinogen. Leptospira interrogans serovar Kennewicki strain Fromm (LPF) supernatant (0.01–0.5 μg total secreted proteins) was incubated with 15 μg of purified fibrinogen (A) or with human plasma containing an equivalent amount of fibrinogen (B) for 2 h at 37°C. Cleavage products were analyzed by SDS- polyacrylamide gel under reducing conditions and gels were silver stained (A) or submitted to Western blot and detected with anti-human fibrinogen (B). *cleavage products. FBG, fibrinogen. [file Image1.TIF]
