# Supplementary material for: Leptospira interrogans Secreted Proteases Degrade Extracellular Matrix and Plasma Proteins From the Host
Source: Front Cell Infect Microbiol. 2018 Mar 27;8:92. doi: 10.3389/fcimb.2018.00092 (PMC5881292; doi:10.3389/fcimb.2018.00092)
Supplement: Supplementary Table 5 — Putative moonlighting proteins identified in LPF and Patoc I culture supernatants. [file Table5.DOCX]

| EMBL GenBank UniProt | Moonlighting proteins  (MoonProt) | Leptospiral culture supernatant |
| --- | --- | --- |
| B0SLJ6 | Catalase-peroxidase (CP) | Patoc I |
| R9A2Z6 | Glutamine synthetase | Patoc I / LPF |
| R9A7H2 | Phosphoglycerate kinase | Patoc I |
| R8ZY22 | Chaperone protein DnaK (HSP70) | Patoc I / LPF |
| B0SMI6 | Serine hydroxymethyltransferase (SHMT) | Patoc I / LPF |
| R8ZYD7 | Aspartate-semialdehyde dehydrogenase | Patoc I |
| V6GWU0 | dCTP deaminase (Deoxycytidine triphosphate deaminase) | LPF |
| R8ZLU6 | ATP-dependent 6-phosphofructokinase (ATP-PFK) (Phosphofructokinase) | Patoc I |
| N1U855 | Thioredoxin | LPF |
| M3IIL3 | Triosephosphate isomerase | LPF |
| Q72PR7 | Isoleucine--tRNA ligase (Isoleucyl-tRNA synthetase) | LPF |
| Q8F7I9 | Glutamate synthase (NADPH) | LPF |
| Q8EYJ8 | Ferredoxin-like sulfite reductase | Patoc I / LPF |
| R9A1K0 | Isocitrate dehydrogenase | Patoc I / LPF |
| M6GAD9 | Fructose-1,6-bisphosphatase class 1 domain protein | LPF |
| M3HY21 | NADH-ubiquinone oxidoreductase-G iron-sulfur binding region | LPF |
| B0SQ31 | Putative alcohol dehydrogenase, zinc-containing alcohol dehydrogenase family | Patoc I / LPF |
| M5ZTQ9 | Transketolase, pyridine binding domain protein | LPF |
| M7A1T9 | Citrate (Si)-synthase domain protein | LPF |
| Q8EXU1 | Peroxiredoxin | Patoc I / LPF |
| Q9XD38 | Elongation factor Tu (EF-Tu) | Patoc I / LPF |
| V6GXM4 | Elongation factor G (EF-G) | Patoc I / LPF |
| Q8F5H7 | Glyceraldehyde-3-phosphate dehydrogenase | Patoc I / LPF |
| M3I747 | Aspartate/ornithine carbamoyltransferase | LPF |
| P61439 | 60 kDa chaperonin (GroEL protein) | Patoc I / LPF |

**Supplementary Table 5.** Putative moonlighting proteins identified in LPF and Patoc I culture supernatants**.**
